# Reliability and correlation of mixture cell correction in methylomic and transcriptomic blood data

**DOI:** 10.1186/s13104-020-4936-2

**Published:** 2020-02-12

**Authors:** Boris Chaumette, Oussama Kebir, Patrick A. Dion, Guy A. Rouleau, Marie-Odile Krebs

**Affiliations:** 1 Department of Psychiatry, McGill University, Montreal, Canada; 2 Université de Paris, Institute of Psychiatry and Neuroscience of Paris (IPNP), INSERM U1266, 102-108 Rue de la Santé, 75014 Paris, France; 3 GHU Paris Psychiatrie et Neurosciences, Hôpital Sainte Anne, Paris, France; 4 CNRS GDR 3557 Institut de Psychiatrie, Paris, France; 5 Montreal Neurological Institute, Department of Neurology and Neurosurgery, McGill University, Montreal, Canada

**Keywords:** DNA, RNA, Leucocytes, Chip, Sequencing

## Abstract

**Objectives:**

The number of DNA methylome and RNA transcriptome studies is growing, but investigators have to consider the cell type composition of tissues used. In blood samples, the data reflect the picture of a mixture of different cells. Specialized algorithms can address the cell-type heterogeneity issue. We tested if these corrections are correlated between two heterogeneous datasets.

**Results:**

We used methylome and transcriptome datasets derived from a cohort of ten individuals whose blood was sampled at two different timepoints. We examined how the cell composition derived from these omics correlated with each other using “CIBERSORT” for the transcriptome and “estimateCellCounts function” in *R* for the methylome. The correlation coefficients between the two omic datasets ranged from 0.45 to 0.81 but correlations were minimal between two different timepoints. Our results suggest that a posteriori correction of a mixture of cells present in blood samples is reliable. Using an omic dataset to correct a second dataset for relative fractions of cells appears to be applicable, but only when the samples are simultaneously collected. This could be beneficial when there are difficulties to control the cell types in the second dataset, even when the sample size is limited.

## Introduction

*Omics* technologies are growing in many biomedical fields. In some of these fields, like psychiatry and neurology, access to tissues of interest is difficult while patients are alive and undergoing evaluation or treatment. Consequently, a number of studies came to rely on blood samples as an alternate source of accessible material from patients [5]. However, DNA methylation and gene expression profiles are relatively specific to a particular tissue and cell types, leading to frequent criticisms in regard to the reliability of results obtained from blood samples. Furthermore, one of the issues associated with the use of blood samples is that these comprised of various cell types. Consequently, the DNA methylation and RNA profiles that are derived from such samples are the results of a mixture of profiles. To detect statistically significant differences in methylation or gene expression data that are related to the experiment design, and not driven by the underlying variability and heterogeneity in cell-type composition, new algorithms have been developed to compute and address this issue. Yet, on occasion such correction cannot be applied because the cell counts composition can only be estimated from a whole-genome *omic* dataset (e.g. methylome and transcriptome) and not from a candidate gene study (e.g. Q-PCR or study of the methylation of one promoter). We want to test if the estimation of cell counts using one modality is reliable to correct the data obtained using a second modality. In a cohort of ten individuals, assessed at two different times (at baseline = T0 and 1-year after = T1), we tested for the correlation of two algorithms (EstimateCellCounts and CIBERSORT) when retrospectively estimating cell counts of methylomic and transcriptomic datasets obtained from the same blood samples. We also tested for the longitudinal stability of the cell counts in the same individuals.

## Main text

The participants were recruited through the ICAAR cohort (PHRC, AOM-07-118, see [3] for a detailed description of the cohort). For the methylomic analysis, genomic DNA (500 ng) was extracted from whole blood, treated with sodium bisulfite using the EZ-96DNA Methylation KIT (Catalog No D5004, Zymo Research, USA) following the manufacturer’s standard protocol. Then the DNA methylation was studied using the Illumina Infinium HumanMethylation450 BeadChip (Illumina, San Diego, CA, USA) which contains 485,000 probes across the genome. The Illumina GenomeStudio software (Illumina, San Diego, CA, USA) was used to assess the signal intensities of each probe. The R Minfi package [2] enabled data quality checks and normalization. This *omic* dataset was previously described in [8]. We used the EstimateCellCounts algorithm [6], which is implemented in Minfi package, to assess the abundances of various cell types in the methylomic dataset: B cells, CD4 T lymphocytes, CD8 T lymphocytes, eosinophils, granulocytes, monocytes and natural killer cells. We decide to not consider eosinophils as their estimates are effectively all zero (Additional file 1: Table S1).

For the transcriptomic analysis, total RNA was extracted from blood samples (PAXgene tubes) following the manufacturer protocol and using the PAXgene Blood RNA kit (QIAGEN) and a QIAcube robot. Then the *omic* dataset was obtained from the sequencing of TruSeq libraries. The methods for RNA sequencing are detailed in Chaumette et al. [4]. Briefly, blood total RNA was processed using the mRNA-Seq Sample Prep Kit (Illumina) before poly(A) RNA was isolated, fragmented and purified by ethanol precipitation. The libraries were prepared using the TruSeq Stranded mRNA kit. Paired-end 75-bp sequencing runs were performed on an Illumina HiSeq 2000 instrument at over 80 million reads per sample. The Illumina software RTA1.12.4.2/HCS1.4.8 converted this fluorophore information to sequence data and obtain FASTA files. Quality control was performed using ShortRead package for R [10]. FASTA files were aligned to the reference genome (hg19) using TopHat2 to generate BAM files [9]. A matrix of read counts was then created using HTSeq [1]. Then we used the CIBERSORT algorithm [11] to assess the abundances of 22 cell types in the transcriptomic data using the gene expression data and the LM22 signature gene file (default file). We only retained, for further analyses, the 6 cell types determined by the EstimateCellCounts algorithm in the methylomic dataset (Additional file 1: Table S1).

Spearman’s and Person’s correlations between cell populations estimated using the methylomic data and the transcriptomic data were tested using SPSS software (IBM SPSS Statistics for Windows, Version 24, IBM Corp., Armonk, NY). The significance threshold was set to an alpha-risk of 0.05 and multiple testing corrections were done using the Benjamini–Hochberg method.

Fractions of cells derived from the methylomic and the transcriptomic datasets were computed and the results are presented in Additional file 1: Table S1. For the comparison between the two modalities, using all the samples, all Spearman’s correlations were significant with a coefficient moderate to high (0.45 to 0.81). The weakest correlation was obtained for CD8 T lymphocytes and the highest for CD4 T lymphocytes (see Table 1). All correlations remained significant after multiple-testing correction by the Benjamini–Hochberg method. Pearson’s correlations are reported in Additional file 1: Table S2. We then tested the correlation in the same individual between T0 and T1. As expected, due to the longitudinal variation of cells, there were fewer significant correlations with weaker coefficients (see Table 1).

**Table 1:** Spearman’s correlation between the proportion of each cell type estimated from the methylomic and transcriptomic datasets and Spearman’s correlation between the proportion of each cell type in T0 and T1 for each dataset

Cell type	Comparison between cell counts obtained from methylomic and transcriptomic dataset (n = 20)			Longitudinal correlation in the methylomic dataset (n = 10)		Longitudinal correlation in the transcriptomic dataset (n = 10)	
	Coefficient	Significance	Significance after BH correction	Coefficient	Significance	Coefficient	Significance
CD8T	0.45	*0.044*	*0.044*	0.79	*0.007*	0.79	*0.006*
CD4T	0.81	*< 10*^*−4*^	*< 10*^*−4*^	0.72	*0.019*	0.81	*0.005*
NK	0.67	*1·10*^*−3*^	*2·10*^*−3*^	0.55	0.100	0.26	0.467
B cell	0.63	*3·10*^*−3*^	*0.005*	0.68	*0.032*	0.52	0.128
Monocytes	0.55	*0.012*	*0.016*	0.82	*0.004*	0.79	*0.006*
Granulocytes	0.76	*0.017*	*0.019*	0.30	0.405	0.21	0.556
Lymphocytes (all)	0.76	*< 10*^*−4*^	*3·10*^*−4*^	0.38	0.276	0.66	*0.038*

*NK* natural killer cells, *CD8T* CD8 T lymphocytes, *CD4T* CD4 T lymphocytes, *B cell* B lymphocytes, *BH* Benjamini–Hochberg

These results indicate that cell heterogeneity can reliably be computed using bioinformatic algorithms like CIBERSORT for transcriptomic data and EstimateCellCounts function in R for methylomic data. Moreover, such a posteriori corrections are easier to apply than a priori adjustment that would involve flow cytometry or microbeads cell separations which are difficult to perform when the samples have been previously frozen.

Given that the correlation between transcriptomic and methylomic data is strong, it is reasonable to consider using the first dataset to predict the cell composition of the second one. This may be particularly useful when the reference-database is not provided for the later or if the second dataset is derived from a non-omic approach. For instance, we can use the cell counts derived from a methylomic dataset to establish a cell mixture composition and then correct the targeted transcriptomic data (e.g. Q-PCR) where the cell counts could not be obtained. Inversely, the genome-wide transcriptome could be used to correct cell counts in a targeted methylation study (e.g. pyrosequencing). Cell composition can be retrospectively estimated and correlated across the different sets of data, even in a dataset with limited sample size, but only when the samples are simultaneously collected. Due to the weak longitudinal correlations, correction of a dataset with the cell mixture composition estimated from another dataset is reliable only when the samples are collected at the same time. It does not appear to be a reliable approach to correct data from one modality when the sample for the second modality has been collected at a different timepoint.

## Limitations

The main limitation of our report is the sample size that is very limited to only 10 individuals. However, the strong correlations obtained demonstrated the high reliability of the algorithms even for small studies. We have to acknowledge that longitudinal correlations are based in 10 samples with 2 timepoints whereas the correlations between the two modalities are based on 20 samples; the smaller sample size may have decreased the significance of the longitudinal correlations.

Only 6 cell types were shared between the two analyses. However, these cell types are those mainly present in blood and worthy to be considered for cell mixture correction in methylomic or transcriptomic analyses.

Another limitation is that we have only performed these examinations from datasets that were derived from human blood. We cannot extrapolate on how significant such correlations would be if other tissues or species had been used. Indeed, the two algorithms used here are anchored to reference-databases of methylomic or transcriptomic profiles obtained from major cell-types deemed to be present in the tissue of interest and uses this reference to infer sample-specific cell-type proportions. Sometimes however the reference of interest is not available for a particular tissue or species, but other algorithms can perform reference-free estimates (e.g. R package RefFreeEWAS for methylomic data) [7].

Finally, it is important to remember that even if some bioinformatics corrections can be applied to the *omic* datasets, the ideal tissue for a specific condition should be extensively discussed. Therefore, additional elements should also be considered when epigenetic and transcriptomic studies are being designed, among which possible batch effect or surrogate variables.

## Supplementary information

**Additional file 1: Table S1.** Raw proportions of the six different cell types both in the methylation dataset and in the RNA sequencing dataset. **Table S2.** Pearson’s correlation between the proportion of each cell type estimated from the methylomic dataset and the transcriptomic dataset.

## Data Availability

Data are available from the corresponding author upon reasonable request.

## Abbreviations

Q-PCR: Quantitative polymerase chain reaction
T0: Baseline
T1: After 1 year of follow-up

## References

[1] Anders S, Pyl PT, Huber W (2015). HTSeq—a python framework to work with high-throughput sequencing data. Bioinformatics.

[2] Aryee MJ, Jaffe AE, Corrada-Bravo H, Ladd-Acosta C, Feinberg AP, Hansen KD, Irizarry RA (2014). Minfi: a flexible and comprehensive bioconductor package for the analysis of infinium DNA methylation microarrays. Bioinformatics.

[3] Chaumette B, Kebir O, Mam-Lam-Fook C, Morvan Y, Bourgin J, Godsil BP, Plaze M, Gaillard R, Jay TM, Krebs MO (2016). Salivary cortisol in early psychosis: new findings and meta-analysis. Psychoneuroendocrinology.

[4] Chaumette B, Kebir O, Pouch J, Ducos B, Selimi F, Gaillard R, Krebs MO, ICAAR Study Group (2019). Longitudinal analyses of blood transcriptome during conversion to psychosis. Schizophr Bull.

[5] Houseman E, Andres Stephanie Kim, Kelsey Karl T, Wiencke John K (2015). DNA methylation in whole blood: uses and challenges. Curr Environ Health Rep.

[6] Houseman Eugene Andres, Accomando William P, Koestler Devin C, Christensen Brock C, Marsit Carmen J, Nelson Heather H, Wiencke John K, Kelsey Karl T (2012). DNA methylation arrays as surrogate measures of cell mixture distribution. BMC Bioinform.

[7] Houseman EA, Molitor J, Marsit CJ (2014). Reference-free cell mixture adjustments in analysis of DNA methylation data. Bioinformatics.

[8] Kebir O, Chaumette B, Rivollier F, Miozzo F, Lemieux Perreault LP, Barhdadi A, Provost S (2017). Methylomic changes during conversion to psychosis. Mol Psychiatry.

[9] Kim D, Pertea G, Trapnell C, Pimentel H, Kelley R, Salzberg Steven L (2013). TopHat2: accurate alignment of transcriptomes in the presence of insertions, deletions and gene fusions. Genome Biol.

[10] Morgan M, Anders S, Lawrence M, Aboyoun P, Pagès H, Gentleman R (2009). ShortRead: a bioconductor package for input, quality assessment and exploration of high-throughput sequence data. Bioinformatics.

[11] Newman AM, Liu CL, Green MR, Gentles AJ, Feng W, Yue X, Hoang CD, Diehn M, Alizadeh AA (2015). Robust enumeration of cell subsets from tissue expression profiles. Nat Methods.

