# Supplementary material for: Reliability and correlation of mixture cell correction in methylomic and transcriptomic blood data
Source: BMC Res Notes. 2020 Feb 12;13:74. doi: 10.1186/s13104-020-4936-2 (PMC7017605; doi:10.1186/s13104-020-4936-2)
Supplement: Supplementary file 1 — Additional file 1: Table S1. Raw proportions of the six different cell types both in the methylation dataset and in the RNA sequencing dataset. Table S2. Pearson’s correlation between the proportion of each cell type estimated from the methylomic dataset and the transcriptomic dataset. [file 13104_2020_4936_MOESM1_ESM.docx]

|  | **Raw proportion of the cell types in the methylation dataset using EstimateCellCount** | | | | | | **Raw proportion of the cell types in the RNA sequencing dataset using CIBERSORT** | | | | | |
| --- | --- | --- | --- | --- | --- | --- | --- | --- | --- | --- | --- | --- |
| **Identifier of the sample** | CD8T | CD4T | NK | B cell | Mono | Gran | CD8T | CD4T | NK | B cell | Mono | Gran |
| IC01-001-T0 | 0.198 | 0.262 | 0.038 | 0.127 | 0.082 | 0.337 | 0.16 | 0.192 | 0.086 | 0.056 | 0.241 | 0.233 |
| IC01-001-T1 | 0.151 | 0.184 | 0.085 | 0.085 | 0.069 | 0.472 | 0.234 | 0.149 | 0.152 | 0.04 | 0.209 | 0.174 |
| IC01-013-T0 | 0.08 | 0.126 | 0.064 | 0.064 | 0.077 | 0.623 | 0.09 | 0.08 | 0.15 | 0.04 | 0.184 | 0.396 |
| IC01-013-T1 | 0.108 | 0.165 | 0.071 | 0.074 | 0.09 | 0.531 | 0.075 | 0.109 | 0.121 | 0.024 | 0.206 | 0.423 |
| IC01-048-T0 | 0.204 | 0.157 | 0 | 0.101 | 0.069 | 0.516 | 0.119 | 0.166 | 0.1 | 0.037 | 0.095 | 0.457 |
| IC01-048-T1 | 0.198 | 0.194 | 0.05 | 0.086 | 0.069 | 0.437 | 0.117 | 0.164 | 0.124 | 0.04 | 0.161 | 0.325 |
| IC01-055-T0 | 0.148 | 0.16 | 0.136 | 0.089 | 0.109 | 0.399 | 0.105 | 0.111 | 0.178 | 0.03 | 0.192 | 0.348 |
| IC01-055-T1 | 0.134 | 0.183 | 0.065 | 0.08 | 0.086 | 0.482 | 0.107 | 0.134 | 0.11 | 0.042 | 0.22 | 0.347 |
| IC01-063-T0 | 0.182 | 0.227 | 0 | 0.123 | 0.066 | 0.439 | 0.086 | 0.261 | 0.083 | 0.068 | 0.068 | 0.395 |
| IC01-063-T1 | 0.112 | 0.198 | 0 | 0.095 | 0.051 | 0.574 | 0.096 | 0.187 | 0.064 | 0.044 | 0.097 | 0.485 |
| IC01-101-T0 | 0.152 | 0.193 | 0.05 | 0.109 | 0.044 | 0.5 | 0.105 | 0.188 | 0.112 | 0.041 | 0.171 | 0.329 |
| IC01-101-T1 | 0.163 | 0.2 | 0.051 | 0.103 | 0.054 | 0.473 | 0.135 | 0.17 | 0.11 | 0.054 | 0.152 | 0.353 |
| IC01-105-T0 | 0.158 | 0.147 | 0.054 | 0.118 | 0.078 | 0.483 | 0.135 | 0.121 | 0.102 | 0.048 | 0.2 | 0.355 |
| IC01-105-T1 | 0.132 | 0.13 | 0.039 | 0.105 | 0.069 | 0.565 | 0.118 | 0.087 | 0.084 | 0.044 | 0.203 | 0.427 |
| IC01-119-T0 | 0.071 | 0.163 | 0.1 | 0.083 | 0.075 | 0.55 | 0.068 | 0.107 | 0.121 | 0.039 | 0.146 | 0.478 |
| IC01-119-T1 | 0.064 | 0.19 | 0.106 | 0.085 | 0.079 | 0.52 | 0.068 | 0.134 | 0.132 | 0.036 | 0.182 | 0.424 |
| IC01-175-T0 | 0.233 | 0.27 | 0.056 | 0.1 | 0.042 | 0.334 | 0.099 | 0.178 | 0.12 | 0.038 | 0.108 | 0.412 |
| IC01-175-T1 | 0.2 | 0.22 | 0.048 | 0.093 | 0.047 | 0.43 | 0.136 | 0.197 | 0.183 | 0.036 | 0.158 | 0.251 |
| IC25-004-T0 | 0.126 | 0.217 | 0.064 | 0.085 | 0.04 | 0.507 | 0.147 | 0.137 | 0.126 | 0.025 | 0.188 | 0.359 |
| IC25-004-T1 | 0.132 | 0.196 | 0.057 | 0.084 | 0.035 | 0.538 | 0.16 | 0.143 | 0.164 | 0.031 | 0.169 | 0.295 |

**Additional file 1: Table S1.** Raw proportions of the six different cell types both in the methylation dataset and in the RNA sequencing dataset.

CD8T: CD8 T lymphocytes; CD4T: CD4 T lymphocytes; NK: natural killer cells; B cell: B lymphocytes; Mono: Monocytes; Gran: Granulocytes
Each individual (identifier ICXX-XXX) is collected either at baseline (T0) or after one year of follow-up (T1).

**Additional file: 1 Table S2**. Pearson’s correlation between the proportion of each cell type estimated from the methylomic dataset and the transcriptomic dataset

| **Cell type** | **Pearson coefficient correlation** | **Significance** |
| --- | --- | --- |
| CD8T | 0.36 | **0.119** |
| CD4T | 0.74 | **2.10^-4^** |
| NK | 0.66 | **2.10^-3^** |
| B cell | 0.72 | **3.10^-4^** |
| Monocytes | 0.42 | **0.068** |
| Granulocytes | 0.47 | **0.037** |
| Lymphocytes (all) | 0.64 | **3.10^-3^** |

CD8T: CD8 T lymphocytes; CD4T: CD4 T lymphocytes; NK: natural killer cells; B cell: B lymphocytes
